# Tracking and treating malnutrition: a retrospective observational study of the nutritional status of vulnerable people accessing a meals-on-wheels (MOW) service

**DOI:** 10.1017/S1463423620000195

**Published:** 2020-06-11

**Authors:** Michelle Dewar, Angela Dickinson, Nigel Smeeton

**Affiliations:** 1 Dietitian, Hertfordshire Independent Living Service, Unit 16, Green Lane One, Blackhorse Road, Letchworth, Herts, SG6 1HB, UK; 2 Senior Research Fellow, Centre for Research in Public Health And Community Care (CRIPACC), Health Research Building, University of Hertfordshire, College Lane Campus, Hatfield, AL10 9AB, UK; 3 Social Statistician, Centre for Research in Public Health And Community Care (CRIPACC), Health Research Building, University of Hertfordshire, College Lane Campus, Hatfield, AL10 9AB, UK

**Keywords:** frailty, independent living, malnutrition, meals-on-wheels, nutrition assessment, older people, support services, undernutrition

## Abstract

**Aim:**

The aims of the study were to describe the characteristics of meals-on-wheels (MOW) recipients, including prevalence of malnutrition amongst those who have received input from the Nutrition and Wellbeing Service (NWS) and to explore whether the NWS had an impact on the nutritional status (malnutrition risk) of recipients over time.

**Background:**

Support services, for example, MOW, play an important role in the prevention and treatment of malnutrition in the community. In the UK, MOW services are under threat. However, little is known about how they support the health and well-being of older people. This study reports on the characteristics of MOW recipients and investigates change in nutritional status over time.

**Methods:**

A retrospective study of MOW recipients of nutritional concern who were offered a check through the NWS was conducted. Demographic, social and health information were gathered at the initial visit. Nutritional status (risk of malnutrition) was obtained using the validated Malnutrition Universal Screening Tool (MUST), at the initial and subsequent visits. Changes over time were investigated for recipients receiving at least two follow-up visits.

**Findings:**

An initial visit was made to 399 MOW recipients, and 148 recipients had two or more follow-up visits. At initial screening, 177 (44%) of recipients were at medium or high risk of malnutrition. Frailty was significantly related to malnutrition risk (*P* = 0.049). At follow-up, there was a reduction in malnutrition risk.

**Conclusions:**

The MOW service was associated with a reduction in malnutrition risk. By offering well-being visits within a MOW service, malnutrition can be identified early. Future studies into how MOW services might delay or prevent the need for support from acute health services and social care are warranted.

## Introduction

Malnutrition is a serious condition, characterised by multifactorial causality (Volkert, 2013), thought to affect 1.3 million (or 1 in 10) older adults in the UK (Malnutrition Task Force, 2013; Malnutrition Task Force, 2019) with 93% of malnutrition occurring in community settings (Elia and Russell, 2009). The prevalence of malnutrition for community-living older adults in Europe and North America varies between 1% and 15% (Favaro-Moreira *et al*., 2016). With a predicted increase in life expectancy within the UK and worldwide (Office for National Statistics, 2017), this global phenomenon of population ageing will result in an increase in the numbers of older people at risk of malnutrition. It is well documented that malnutrition in older adults is associated with poor clinical outcomes and increased health and social care use (Agarwal *et al.*, 2013). The costs associated with malnutrition within England are substantial, estimated at £19.6 billion per year (£23.5 billion in the UK), equivalent to around 15% of total health and social care (Elia, 2015). Despite these statistics, there is little academic work exploring interventions to either prevent or address malnutrition in older people.

Meals-on-wheels (MOW) have played an important part in supporting older people to remain food secure in their own homes in the UK since World War II, providing nutrition, food security and social contact (Winterton *et al.*, 2013; Campbell *et al.*, 2015). However, in recent years, as a result of austerity impacting on local authority budgets, MOW across the UK have been in decline (Sustain, 2013; Mortimer and Green, 2016). A report by Sustain (2013) expressed concern that older people may not be receiving the food services and associated care that they need. This is supported by Walton et al. (2020) who stated that factors relating to the inability of providing for oneself, such as access, preparation or mobility concern, are usual requirements to access a service like MOW, and an inability to access sufficient food to meet nutritional needs may be related to malnutrition in older people. The decline in MOW provision has coincided with a significant increase in the number of malnutrition-related hospital admissions (Malnutrition Task Force, 2017). There is some evidence that people receiving MOW have better mental health and fewer falls (Thomas *et al*., 2016); reduced hospital admissions (Luscombe-Marsh *et al.*, 2014; Cho *et al.*, 2018); shorter hospital stays (Cho *et al*., 2018); feel less lonely and have improved well-being (Wright *et al*., 2015).

Hertfordshire Independent Living Service (HILS) is a thriving social enterprise serving up to 5000 MOW recipients annually. HILS offers three meal services (breakfast, lunch and tea). All recipients receive the lunchtime service which is delivered hot each day, 365 days of the year. Meals are dropped off and can be plated up for the recipient and a drink made. The worker does not stay and eat with the recipient. Recipients can choose to receive additional meal services, including breakfast and/or tea service and these are delivered (cold) at the same time as the hot lunch. The majority of meal recipients receive the lunch service only and make a financial contribution towards the meals.

Over half (59%) of MOW providers offer additional services such as well-being checks and signposting to other services (Dickinson *et al*., 2016; Association for Public Service Excellence on behalf of the National Association of Care Catering, 2018). However, HILS is unique in offering a Nutrition and Wellbeing Service (NWS), established in 2014, that aims to identify, treat and prevent malnutrition. The service offers a nutrition and well-being screening visit by a member of the HILS staff to all vulnerable MOW recipients experiencing risk factors for malnutrition. Risk factors include unintentional weight loss, low body weight, swallowing difficulty, poor appetite or other nutritional or well-being concern. Referrals are received from recipients, their families, other referrers or HILS staff, who are trained to check for signs of malnutrition and dehydration. The NWS assessment involves a series of questions around nutrition, social circumstances (e.g., whether a recipient lives alone), health and well-being (including appetite, fluid intake, mobility, frailty, loneliness and mood, sensory concerns) and weight and height measurements to assess for malnutrition.

Following the NWS visit, recipients receive a range of appropriate interventions, tailored to individual need, such as provision of extra nutrition by including higher energy meals, energy dense mini-meals or texture-modified meals. High calorie snacks are provided free of charge. Education about food fortification, educational literature and practical items, such as placemats with nutritional messages and water-jugs promoting fluid intake, are provided. Referrals are made to other providers if any issues are identified, to address social, health and well-being issues, such as incontinence, mental health and sensory issues. In-house dietetic support involving an additional visit and more tailored dietetic plan is offered to those at greatest nutritional risk.

Programmes such as MOW potentially provide substantial savings to healthcare (Thomas and Mor, 2013) and are well placed to refer to support services to help people remain independent (Polzer, 2017; Volkert *et al*., 2019). One Australian study (Walton *et al*., 2015) and a systematic literature review that included 13 studies (Walton *et al*., 2020) found that MOW may be an important contributor to the overall intakes of energy, protein and some micronutrients compared to when meal services are not received. This may have a positive influence on malnutrition risk. Walton *et al.* (2015) suggested a need for regular screening and monitoring in this vulnerable at-risk group. We know very little about MOW recipients living in the UK with the most recent UK-based academic literature stemming from the early 1980s (Campbell *et al.*, 2015). This paper addresses a significant gap in the UK literature by reporting on data collected routinely by the NWS service at HILS. The aims of the study were to describe the characteristics of MOW recipients, including prevalence of malnutrition amongst those who have received input from the NWS service and explore whether the NWS had an impact on the nutritional status (malnutrition risk) of recipients.

## Materials and methods

### Recipients

The study population consisted of community-living adults residing in Hertfordshire, UK, receiving a MOW service from HILS and who had received a NWS visit.

Recipients were advised about how the anonymous data may be used, to improve the service and for research purposes. Recipients who did not want their data to be used in this way were excluded from this retrospective study.

Following the initial review visit, recipients were offered a well-being review visit within three to six months depending on the malnutrition risk identified, and a subjective judgement by the assessor whereby malnutrition risk factors such as poor appetite or borderline healthy body mass index (BMI) were noted. Data from additional interim visits such as weight checks or dietetic reviews have not been included in the analysis. The time between review visits varied between recipients according to their needs and the subjective judgement of the assessor. Some visits were postponed due to hospitalisation or similar reasons.

This paper reports on the characteristics of the recipients at the initial review visit. In addition, the study incorporated a longitudinal element. All individuals who received at least two follow-up reviews, based on the malnutrition risk identified, over a minimum period of six months were included in the study cohort sub-sample of recipients. A more stringent criterion for selection was avoided in order to maximise retention in the original cohort.

Data included in the study were collected between December 2015 and March 2018.

### Sample

All recipients received at least one nutrition and well-being visit from a HILS Dietitian, Nutritionist or Nutrition and Well-being Visitor. All staff members involved in well-being checks have been trained in how to assess for malnutrition and have undergone a period of supervision by a registered nutritionist or dietitian. The visit involves gathering data about recipients’ current health and well-being by asking a series of questions and collecting measurements of weight and height, as well as demographic information and questions relating to social circumstances. Information was recorded in paper-based form and transferred to an electronic database reflecting the questions on the form. The database was designed to meet the needs of the service and to enable the capture of change of nutritional status over time at subsequent reviews.

Nutritional status was assessed using the Malnutrition Universal Screening Tool (MUST) (BAPEN, 2011). There are three steps used within this tool to identify malnutrition risk. These are based on BMI, recent unintentional weight loss and acute disease effect. This tool is validated and is commonly used in both acute and community settings (Todorovic *et al*., 2011).

BMI was calculated using weight and height measurements. Weights were recorded using Marsden floor scales (model M-540) that are class III medically approved as suitable for healthcare settings, placed on a flat surface for weighing. Actual weights were used for most recipients (92% at initial visit), with alternative measures, such as reported or estimated weights used for a minority of cases (2% at initial visit). Height was recorded from actual height taken at the visit where appropriate using the portable Marsden Leicester height measure, ulna measurements or height as reported from the recipient, if deemed accurate. Alternative measurements, such as reported weight or mid upper arm circumference, were used to calculate BMI when measuring weight was not possible (6% at initial visit).

Where weight and height measurements were not possible (two people in this sample), self-reported measurements, surrogate measurement and clinical judgement were used to estimate overall malnutrition risk; this is deemed a reliable alternative (Todorovic *et al*., 2011).

When calculating weight loss score at the initial visit, a previous weight was obtained from either recipient report or estimated using subjective measures. When completing follow-up visits, the weight from the previous visit was used to calculate weight loss in subsequent reviews unless the recipient had not been weighed at the previous visit.

This study reports on the change in nutritional status over time by considering the MUST risk at the initial visit and comparing this with the MUST risk from the most recent visit. Of all review assessments, six recipients received a telephone review on one occasion where information collected over the phone was used to estimate MUST score (0–4). Telephone assessment was only offered if a recipient had been under review for a minimum of 15 months, had received at least three prior visits and where the clinician involved had no concerns about their nutritional status.

The MUST tool looks at the acute disease effect, which considers if the person is acutely ill and there has been or is likely to be no nutritional intake for more than five days. This was omitted from the assessment as all recipients were community living (BAPEN, 2012).

Recipients were assessed for frailty at the initial visit. The PRISMA 7 questionnaire was chosen as the most feasible tool for measuring frailty in older community-living adults. This tool was thought to be the safest, easiest and most accurate for the service (Hoogendijki *et al*., 2013). This is a seven-item self-reported questionnaire which considers age, gender, health issues, mobility and support needs. A score of three or more out of a possible total of seven is considered to identify frailty.

The 1-hour visit prioritised obtaining information to assess malnutrition to ensure that the service provision was suitable for nutritional needs. If a recipient was found to be at risk of malnutrition, the principle of increasing daily intake by at least 500 kcal was applied. The recipient was advised of a number of methods in which this could be achieved. This included highlighting meals offered on HILS menu providing the most energy (main meals and desserts). Calorie content of main meals offered by HILS varies between 300 and 600 kcal and dessert varies from 60 to 370 kcal.

Recipients were offered additional foods called ‘Nutrition Boost’ free of charge with the aim to add additional energy (snack items between 150 and 500 kcal such as nutritious soups, cream tea scones, milkshake drinks and high-energy snacks). Recipients were also provided with an information booklet which illustrated easy ways to fortify and enrich meals and drinks or how to make easy swops to add extra energy. Those at risk were also offered a review visit within a three-month period.

Recipients were also asked questions about their health and well-being during the home visit as well as basic demographic information.

Deprivation was calculated using the recipient’s postcode with the English Index of Multiple Deprivation (Ministry of Housing, Communities and Local Government, 2015). Scores were converted to deciles with the most deprived decile indicated by 1 and the most affluent decile by 10.

## Data analysis

Data were exported from the MOW nutrition database onto Microsoft Excel and re-coded for analysis (personal identifying data were not transferred). Data were analysed using IBM SPSS Statistics, version 25 (IBM Corp., Armonk, NY, USA).

The variables of interest were those likely to be both reliable and consistently reported at the initial well-being visit. As well as malnutrition risk, these were gender, age, deprivation index, being able to count on somebody for support, regular use of a stick, walker or wheelchair, falls in the last six months, and frailty (PRISMA-7).

To investigate associations with malnutrition risk, univariate analyses were performed on each variable in turn. Proportions were compared using the chi-squared test. Ordered and quantitative variables were analysed using Kruskal–Wallis analysis of variance and one-way analysis of variance.

Multinomial logistic regression (Hosmer *et al*., 2013) was performed to investigate multivariable relationships associated with malnutrition risk.

## Results

Of the study participants, two-thirds (66%) of the recipients accessing input from the nutrition service received lunchtime meals every day (seven days per week) with an additional 30% receiving meals at least three to six days per week. Most of the recipients (87%) received the lunchtime service only. In addition to the lunch service, around 12% received the tea service, less than one per cent received the breakfast service, less than one per cent received all three services.

Baseline characteristics were collected from 399 recipients (259 females and 140 males) at the initial visit (Table 1). Mean (SD) age of recipients was 83.4 (10.9) years. Around three quarters of recipients were aged over 80 years, living alone and identified as being frail using the PRISMA 7 classification. Information regarding recent falls was provided by 350 recipients with almost half having suffered a fall within the six months prior to their initial visit; 61% reported regular use of a mobility aid to get about. Regarding baseline nutritional status, 56% of recipients were at low risk of malnutrition and 44% at medium or high risk at the initial visit.

**Table 1.** Characteristics of MOW recipients (initial visit) (number of cases with data available, *n,* is 399 unless otherwise stated)

Characteristic	*Frequency*	Prevalence, %
Gender		
Males	140	35
Females	259	65
Age		
Average (mean) age in years (SD)	399	83.4 (10.9)
Recipients aged 80 or older	294	74
Recipients aged 90 or over	124	31
Lives alone	295	74
Can count on somebody close	347	87
Can count on somebody, geographically close (*n* = 347)	296	85
Deprivation (*n* = 213)		
Lives in more deprived area (areas 1–3)	28	7
Lives in the least deprived areas (areas 8–10)	185	46
Regularly uses a stick, walker or wheelchair to get about	244	61
Had a fall in last six months (*n* = 350)	169	48
Drinking <6 drinks per day (*n* = 303)	133	44
Urine infection in last six months	93	23
Incontinence urgency or frequency concern	187	47
Frailty (PRISMA 7)	301	75
Malnutrition risk (MUST)		
High risk	100	25
Medium risk	77	19
Low risk	222	56

Of the review participants, 70% received lunchtime meals every day (seven days per week) with an additional 26% receiving meals at least three to six days per week. Most of the recipients (85%) received the lunchtime service only. In addition to the lunch service, around 15% received the tea service, less than one per cent received the breakfast service, less than one per cent received all three services.

The 148 review participants comprised 90 females and 58 males. Mean (SD) age was 81.6 (11.6) years. Around 65% were aged over 80 years, 70% were living alone and 75% were identified as being frail using the PRISMA 7 classification. Around 40% had experienced a fall within the six months prior to their initial visit; 60% reported regular use of a mobility aid to get about. Regarding baseline nutritional status, 57% of recipients were at low risk of malnutrition and 43% at medium or high risk at the initial visit.

### Relationships with malnutrition risk

Baseline recipient characteristics are shown in Table 2. In the univariate analyses, risk of malnutrition was associated with frailty (*P* = 0.049). No other significant univariate relationships were observed so multivariable analyses were not performed.

**Table 2.** Association of recipient characteristics with malnutrition risk (Number of cases, *n,* is 399*)

	Risk of malnutrition (%)			
	Low *n* = 222	Medium *n* = 77	High *n* = 100	*P*-value
Female (the remaining percentage male)	64	58	71	0.216
Mean age (SD)	82.7 (11.5)	83.8 (11.1)	84.6 (8.7)	0.344
Lives alone	72	79	74	0.469
Deprivation [median score, (IQR)]	7 (5, 9)	8 (5, 10)	7 (5, 9)	0.384
Regularly uses a stick, walker or wheelchair to get about	59	64	64	0.615
Had a fall in the last six months (*n* = 350)	46	43	58	0.104
Drinking <6 drinks per day (*n* = 303)	39	47	53	0.110
Urine infection in the last six months	24	19	25	0.660
Incontinence concern	51	48	36	0.037
Frail (PRISMA 7)	71	82	81	0.049
Frail (PRISMA 7) Male (*n* = 140)	76	88	79	0.397
Frail (PRISMA 7) Female (*n* = 259)	68	78	82	0.074

*unless otherwise stated.

Source: Multinomial logistic regression did not change the statistical significance/non-significance of these variables.

### Nutritional status over time

Review recipients (*n* = 148) consisted of all who, based on the malnutrition risk identified, received at least two follow-up reviews over a minimum period of six months. The percentage of recipients at low risk within this review group (57%) was like that for those not followed up (55%). This similarity between the two groups seems surprising. However, the 274 clients who stopped receiving the MOW service during the study period did so for a range of reasons, some negative and some positive. Almost one quarter (23%) of those who discontinued did so because of an admission to hospital whereas a similar proportion (23%) left because they felt that they could manage without the MOW service.

Recipients in the review group were similar in other respects compared to the remainder apart from being younger (mean age 81.6 versus 84.4, 95% CI for difference 0.5 – 5.0, *P* = 0.018) and being less likely to have had a fall in the previous six months (40% versus 53%, *P* = 0.023).

Considering recipients as three categories according to malnutrition risk (low, medium and high), over 90% of recipients (low risk 80/85, medium risk 23/25, high risk 35/38) remained stable or improved their malnutrition risk at the most recent review within each risk group. Change in prevalence of malnutrition risk between initial and most recent review is detailed further in Table 3.

**Table 3.** Change in risk of malnutrition over time (Number of cases, *n*, is 148)

Risk of malnutrition	Recipients (%) at initial visit	Malnutrition risk change for those in this category at the initial visit			Recipients (%) at most recent review
		Improved	Stable	Worsened	
Low (score 0)	85 (57)	Not applicable	80 (94)	5 (6)	108 (73)
Medium (score 1)	25 (17)	19 (76)	4 (16)	2 (8)	14 (9)
High (scores 2–4)	38 (26)	23 (60.5)	12 (31.5)	3 (8)	26 (18)

## Discussion

This is the first UK-based study that reports on the characteristics of community-living people receiving a combination of a MOW service and NWS, as well as assessing and reporting on change in nutritional status (malnutrition risk) over time following input from a bespoke NWS.

Most of the MOW recipients, whether viewed as a whole or considering the review recipients only, were older adults and predominantly female, which is comparable to other studies of people receiving or waiting for MOW (Frongillo *et al*., 2010; Polzer, 2017; Thomas *et al*., 2017). This finding is unsurprising given the gender ratio in the older population (Office for National Statistics, 2018). Recipients were more likely to live alone (Polzer, 2017) than the general older population in the UK (Office for National Statistics, 2018) and this echoed studies in other countries (Frongillo *et al*., 2010). Living alone is a risk factor for social isolation (Office for National Statistics, 2018); however, most MOW recipients reported having somebody in their lives they could rely on, which differed from a USA-based study (Frongillo *et al*., 2010).

Interestingly, few recipients lived in the more deprived areas of Hertfordshire, which poses the question as to whether recipients living in these areas are less likely to self-refer or be referred to the service. This may be due to having a poor support network, lack of knowledge of the service or perhaps being less likely to accept being seen by the service. Further research is required to determine to what extent health inequalities affect access to MOW services.

This study found that MOW recipients had poorer mobility than that of a USA-based study (Frongillo *et al.*, 2010) and the general older UK population (Malnutrition Task Force, 2017), and this could be due to those with nutritional concerns self-referring or being referred by their support network to NWS. Poor mobility, as well as social factors, such as loneliness and bereavement, affects food security through limiting the ability to shop for food and can reduce motivation to eat and drink well (Malnutrition Task Force, 2017).

Frailty is associated with an increased risk of adverse health outcomes (Volkert *et al*., 2019) and is an important determinant and risk factor for malnutrition (Favaro-Moreira *et al*., 2016; Chang, 2017). With around 75% of MOW recipients meeting the PRISMA 7 frailty criteria, the NWS service is well placed to identify and possibly address frailty promptly, offer support to promote and maintain independence and to prevent malnutrition through regular monitoring and support. Frailty was not measured during subsequent visits and therefore we cannot measure any impact that the service has on frailty, though this would be useful for further studies in this population. Prevalence of frailty within this study (75%) was higher than the 10–65% reported in other studies of the general UK population of older people (Clegg *et al*., 2013; Gale *et al.*, 2015). However, a study in Australia found that frailty in men is associated with the use of health and community services, such as support with meals (Rochat *et al*., 2010) which may help explain the higher prevalence reported within this study.

Although the pathophysiology of malnutrition and frailty share common pathways (Verlaan *et al*., 2017), they are distinct conditions (Wei *et al.*, 2017). Whilst different tools have been used to assess frailty and malnutrition, a recent systematic review found that these conditions are significantly related to one another (Verlaan *et al*., 2017), which supports the significant relationship reported within this study.

Groups at risk of malnutrition include older adults, frail adults and those requiring support with meals (Malnutrition Pathway, 2017), and this MOW population has multiple risk factors. All community-living older adults should be routinely screened for malnutrition (Volkert *et al*., 2019), and the updated UK community malnutrition pathway (Malnutrition Pathway, 2017) suggests opportunistic screening, using a validated tool such as MUST, for example, at first contact in a new setting, attendance at an outpatient appointment and other opportunities within the community. Where malnutrition is common, the routine use of a simple screening tool is recommended (Elia *et al.*, 2005). A review by Agarwal *et al*. (2013) on malnutrition in older people suggested that routine nutritional screening is important in identifying and treating malnutrition in a timely fashion. Individualised nutritional support to malnourished individuals can result in positive outcomes. With 44% of recipients identified as at risk or suffering malnutrition, this study suggests that screening should be offered to all those in receipt of MOW. This study shows a greater prevalence of older adults suffering from or at risk of malnutrition within the MOW population than in the wider UK older adult population (Malnutrition Task Force, 2017), and this is comparable to the limited international studies reporting specifically on MOW recipients (O’Dwyer *et al.*, 2009; Walton *et al.*, 2015). The Malnutrition Task Force (2017)suggested that prevalence of malnutrition may be much higher than the current figures reported, and this may be due to a lack of widespread population screening. This study supports this hypothesis.

### The effectiveness of MOW in addressing malnutrition

Baseline nutritional status of review recipients was comparable to the original sample suggesting that the review group was broadly representative of the whole group. Over 90% (138/148) of recipients receiving the NWS service maintained or improved their nutritional status over time and the proportion of recipients identified by MUST as being at low risk of malnutrition improved noticeably from 57% to 73%. The authors are unaware of any other services that offer a similar NWS for MOW recipients in the UK. The NWS is unique and differs from interventions previously reported in that it offers an enhanced service to vulnerable people receiving MOW. The NWS offers an extensive meal service, for example, higher energy or texture modified meals with regular nutritional screening, free-of-charge high-energy snacks, ongoing monitoring and dietetic input as required, as well as onward referrals to address non-nutrition health and well-being issues. By offering this enhanced service, nutritional status has been maintained or improved for many recipients receiving the enhanced service. Whilst this study shows that nutritional status has been maintained or improved in those receiving an enhanced service, it cannot conclude that this success is due to the enhanced service alone without knowing more about any other support services that a recipient may have been receiving. An intensive food-based approach is supported by the systematic review of Hamirudin *et al.* (2016) and the recent ESPEN nutrition and hydration guidelines for older people (Volkert *et al*., 2019) who suggest that timely identification of malnutrition risk using a validated screening tool together with appropriate interventions and ongoing monitoring improves the nutritional status of community-living older adults.

## Strengths and limitations

The strength of the present study lies in the uniqueness of the data set; detailed data of this vulnerable MOW population have not previously been reported in the UK. The study provides a greater understanding of the characteristics of the recipients of a bespoke NWS who are receiving MOW. It also reports on longitudinal data, to enable an understanding of the positive impact of a NWS in association with MOW and the effects of an enhanced person-centred meal service on nutritional status over time.

When using a tool such as PRISMA 7 to assess frailty, it is recommended that a second measure, such as gait speed, should be used in order to improve accuracy, but this requires further investigation (Turner, 2014). However, these secondary assessments were not practical for this population due to limited time available for assessments, space and safety concerns within the recipient’s home.

The data around recipient characteristics were collected during the initial visit so only provide a cross-sectional analysis of that time. Further studies looking at these variables and how they may change over time are warranted. In addition, qualitative studies that explore older people’s experiences of both MOW and NWS are warranted.

This study reports on a small proportion of people receiving MOW, who were in receipt of additional input from the NWS; therefore, characteristic trends reported may not be applicable to the whole MOW population. However, it does provide useful insight into the vulnerability of recipients presenting to the NWS.

This study is limited in that there was no control group with which to compare the MOW recipients and therefore direct causality cannot be assessed. There may also have been confounding factors about which no information was available that could have influenced the findings.

## Conclusions

The NWS clearly provides an innovative and proactive service. This study highlights how a NWS operating within a MOW-providing organisation is able to support recipients receiving MOW. Offering a service of tailored nutritional provision, regular screening and monitoring enabled maintenance and improvement of nutritional status over time. However, the current study cannot provide direct causality, but makes a case for further investigation of the impact of the NWS. Any further decline in MOW services in the UK is of concern, given the potential of MOW and an enhanced service such as the NWS to play a role in preventing and alleviating malnutrition in older people.
